# Bimodal CNN for cardiovascular disease classification by co-training ECG grayscale images and scalograms

**DOI:** 10.1038/s41598-023-30208-8

**Published:** 2023-02-20

**Authors:** Taeyoung Yoon, Daesung Kang

**Affiliations:** Department of Healthcare Information Technology, Inje University, Inje-ro, Gimhae-si, 50834 Republic of Korea

**Keywords:** Biomedical engineering, Arrhythmias

## Abstract

This study aimed to develop a bimodal convolutional neural network (CNN) by co-training grayscale images and scalograms of ECG for cardiovascular disease classification. The bimodal CNN model was developed using a 12-lead ECG database collected from Chapman University and Shaoxing People's Hospital. The preprocessed database contains 10,588 ECG data and 11 heart rhythms labeled by a specialist physician. The preprocessed one-dimensional ECG signals were converted into two-dimensional grayscale images and scalograms, which are fed simultaneously to the bimodal CNN model as dual input images. The proposed model aims to improve the performance of CVDs classification by making use of ECG grayscale images and scalograms. The bimodal CNN model consists of two identical Inception-v3 backbone models, which were pre-trained on the ImageNet database. The proposed model was fine-tuned with 6780 dual-input images, validated with 1694 dual-input images, and tested on 2114 dual-input images. The bimodal CNN model using two identical Inception-v3 backbones achieved best AUC (0.992), accuracy (95.08%), sensitivity (0.942), precision (0.946) and F1-score (0.944) in lead II. Ensemble model of all leads obtained AUC (0.994), accuracy (95.74%), sensitivity (0.950), precision (0.953), and F1-score (0.952). The bimodal CNN model showed better diagnostic performance than logistic regression, XGBoost, LSTM, single CNN model training with grayscale images alone or with scalograms alone. The proposed bimodal CNN model would be of great help in diagnosing cardiovascular diseases.

## Introduction

Cardiovascular diseases (CVDs) can cause heart attacks, strokes, and death by causing plaque to build up in the arteries of the heart, in the brain, and block inside important blood vessels in the body. This coronary heart disease is the leading cause of death in the United States^1^. There are various methods for diagnosing CVDs using MRI, X-ray, ultrasound, and heart sounds^2–6^. Among many methods, the clinical tool primarily used to diagnose the heart conditions is the standard 12-lead electrocardiogram (ECG) because it is simple, non-invasive, and inexpensive. However, interpretation of ECG is time consuming and requires highly trained and experienced cardiologists. Computed aided detection and classification of cardiac abnormalities can help cardiologists make an accurate diagnosis.

Many recent attempts have been made to automatically detect and classify CVDs in ECG using machine learning and deep learning techniques^7–14^. For the machine learning techniques, various morphological and statistical features of ECG signals are manually extracted in the time domain, frequency domain, or nonlinear domain, and then the features are applied to machine learning algorithms such as support vector machines, k-nearest neighbors and linear discriminants^7,9,10^. On the other hand, deep learning techniques learn in an end-to-end manner by automatically extracting features from the data. The most commonly used deep learning methods for processing one-dimensional signals such as ECG are recurrent neural networks (RNN), long short-term memory (LSTM), and gated recurrent units (GRU)^11,12^. In addition to RNN-family methods, ECG signals can also be classified by applying convolutional neural networks (CNN) methods such as 1D-CNN and 2D-CNN^8,13,14^. 1D-CNN is relatively easy to train and offers minimal computational complexity while achieving state-of-the-art performance levels. It is especially suitable for mobile or handheld devices with limited computational power and battery life^15^. In order to apply an ECG signal to 2D-CNN, a one-dimensional signal must be converted into a two-dimensional signal. In general, a method of converting a one-dimensional signal into a two-dimensional signal includes (1) plotting the one-dimensional signal itself in two dimensions, (2) transforming the signal into a spectrogram or a scalogram^16,17^. 2D-CNN approaches to classifying CVDs have mainly focused on applying only grayscale images or scalograms.

However, recent studies have shown that the performance of multi-modal, multi-view or multi-input deep learning approaches can be better than that of single-input deep learning approaches^18–20^. To take advantage of the multi-modal deep learning approaches, ECG grayscale images and scalograms were used as inputs in this study. ECG grayscale image can provide intuition to identify CVDs. Cardiologists make a diagnosis by observing the patient's ECG graph on a monitor. The ECG graph used by cardiologists is similar to an ECG grayscale image. This means that the deep learning model is able to learn the cardiologist's knowledge from ECG grayscale images. On the other hand, scalogram has the advantages of automatically removing noises such as baseline wandering effect, power line interference, EMG noise and artifacts. In addition, scalogram can analyze signals jointly in time and frequency on multiresolution and provide more interpretable results. In this study, we aim to improve the performance of CVDs classification by developing a bimodal CNN model that takes advantage of both ECG grayscale images and scalograms.

The main contributions of this study can be summarized as follows:

1. To the best of our knowledge, we first developed a bimodal CNN model for CVDs classification by jointly training ECG grayscale images and scalograms.
2. We showed that the proposed bimodal CNN architecture can be applied to other CNN models through ResNet-50 and EfficientNet-B3.
3. The proposed bimodal CNN model achieved better performance than logistic regression, XGBoost, LSTM and single CNN models for classifying CVDs.

The rest of this study is organized as follows. “Results” section provides the experimental findings obtained by the proposed bimodal CNN model. After “Results” section, the comprehensive summary of our study is described in “Discussion” section. “Conclusion” section presents the conclusion of the proposed method. In “Materials and methods” section, we represent the dataset, preprocessing, details on the architecture of the proposed bimodal CNN model, and performance metrics.

## Results

**Figure 1:**
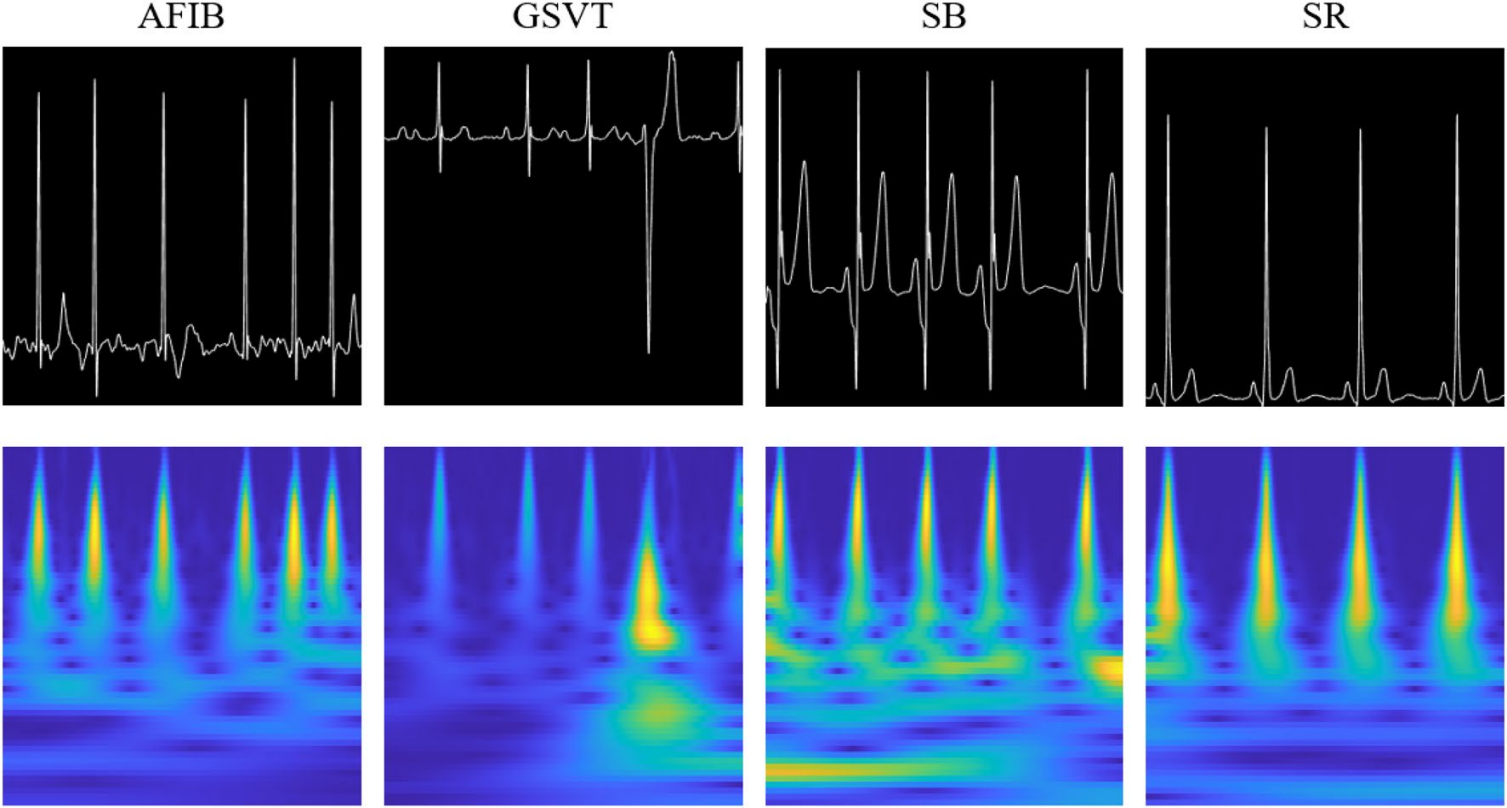
Sample images of grayscale images and scalograms for the four groups of ECG recordings (AFIB, GSVT, SB, and SR). Each column represents the AFIB, GSVT, SB, and SR classes. The first row is grayscale images of the ECG signals, and the second row is scalograms of the ECG signals.

We used 11 ECG rhythms dataset collected by Chapman University and Shaoxing People’s Hospital (Shaoxing Hospital of the Zhejiang University School of Medicine)^21^. The 11 ECG rhythms were hierarchically merged into four groups (AFIB, GSVT, SB, and SR) as suggested by Zheng^21^. The characteristics on the dataset are summarized in Table 1 and sample images of grayscale images and scalograms for the four groups of ECG recordings are shown in Fig. 1.

**Table 1:** Information on the 11 ECG rhythms and the 4 merged ECG groups.

11 ECG groups	Number of subjects	(Merged) 4 ECG groups	Number of subjects
Atrial Fibrillation (AFIB)	1780	AFIB	2218
Atrial Flutter (AF)	438		
Atrial Tachycardia (AT)	121	GSVT	2260
Atrioventricular Node Reentrant Tachycardia (AVNRT)	16		
Atrioventricular Reentrant Tachycardia (AVRT)	8		
Sinus Atrium to Atrial Wandering Rhythm (SAAWR)	7		
Sinus Tachycardia (ST)	1564		
Supraventricular Tachycardia (SVT)	544		
Sinus Bradycardia (SB)	3888	SB	3888
Sinus Rhythm (SR)	1825	SR	2222
Sinus Irregularity (SI)	397

**Figure 2:**
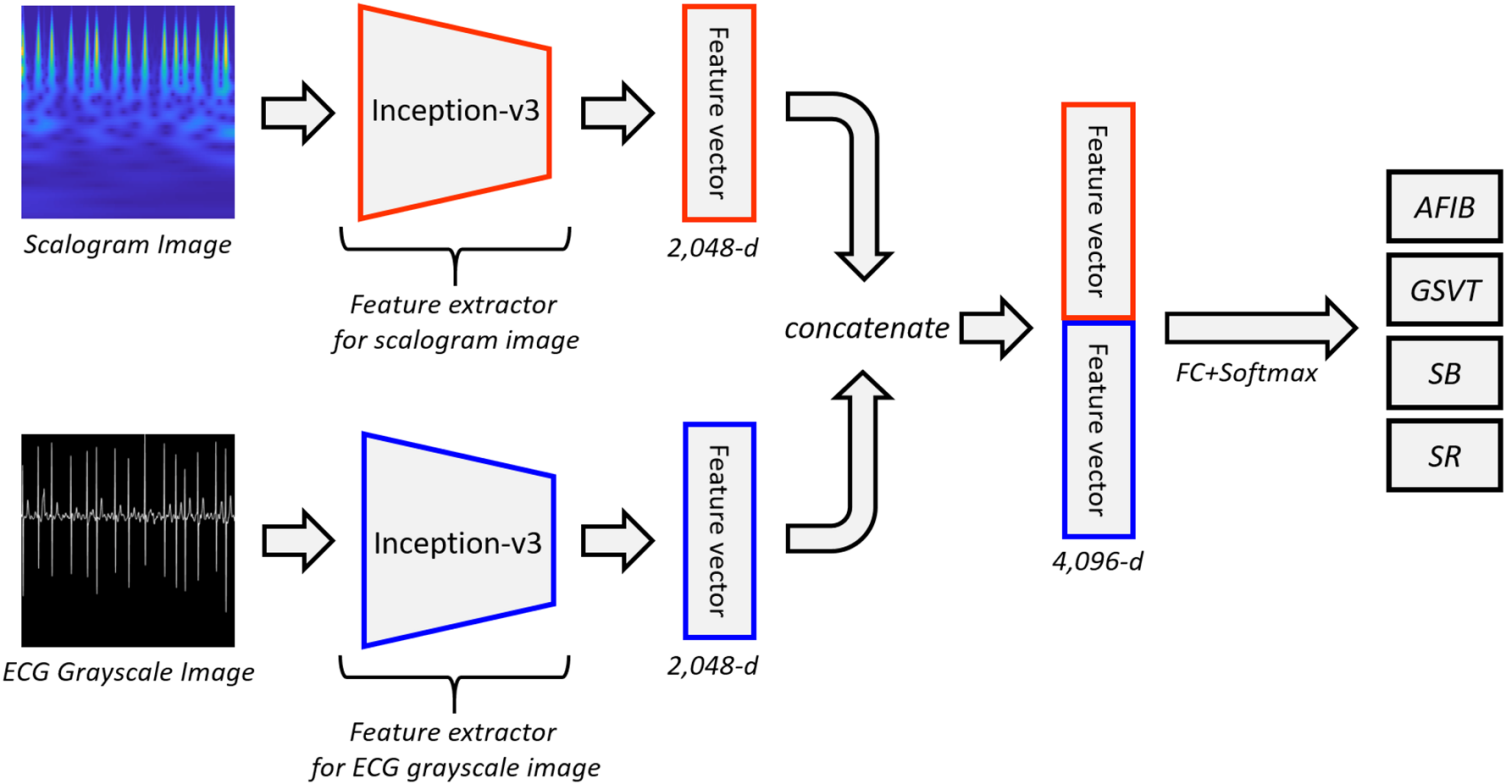
Proposed bimodal CNN model architecture. Grayscale images and scalograms are fed to identical Inception-v3 models simultaneously. Output features of the two Inception-v3 are concatenated and fed into a fully connected classification layer with a softmax activation function that outputs prediction values in the range of 0–1 for the 4 labels.

Table 2 shows the diagnostic performance of a bimodal CNN model with two identical Inception-v3 backbones as described in Fig. 2. The Inception-v3 model is one of the well-known CNN model that scales up networks through suitably factorized convolutions and aggressive regularization^22^. We used a learning rate of 1e−4 during training phase because the learning rate had better accuracy than 1e−5 and 5e−5 for the validation dataset. The diagnostic performance for learning rates of 1e−5 and 5e−5 were shown in the supplementary materials (Table S1, Table S2). Of the 12 leads, lead II achieved best AUC (0.992), accuracy (95.08%), sensitivity (0.942), precision (0.946), and F1-score (0.944). An ensemble model was generated by averaging the cardiovascular disease probabilities for each lead which resulted in better AUC (0.994), accuracy (95.74%), sensitivity (0.950), precision (0.953), and F1-score (0.952). To assert that the bimodal CNN model is valid not only for the two identical Inception-v3 backbones model but also for other backbone models, we included diagnostic performance for the two identical ResNet-50 backbones and two identical EfficientNet-B3 backbones in the supplementary materials (Table S3, Table S4).

**Table 2:** Diagnostic performance of bimodal CNN model for all leads (learning rate = 1e−4).

Lead names	AUC	ACC (%)	SEN	PRE	F1-score
Lead I	0.987	93.19	0.921	0.926	0.922
Lead II	0.992	95.08	0.942	0.946	0.944
Lead III	0.991	94.65	0.937	0.941	0.939
aVR	0.992	94.18	0.932	0.940	0.936
aVL	0.989	93.76	0.929	0.931	0.93
aVF	0.987	93.38	0.924	0.929	0.925
V1	0.99	94.99	0.942	0.946	0.943
V2	0.987	93.09	0.920	0.925	0.921
V3	0.985	92.86	0.917	0.922	0.919
V4	0.985	93.38	0.923	0.927	0.924
V5	0.985	93.05	0.919	0.924	0.920
V6	0.985	92.01	0.907	0.914	0.908
Ensemble	0.994	95.74	0.950	0.953	0.952

ACC, accuracy; SEN, sensitivity; PRE, precision.

**Figure 3:**
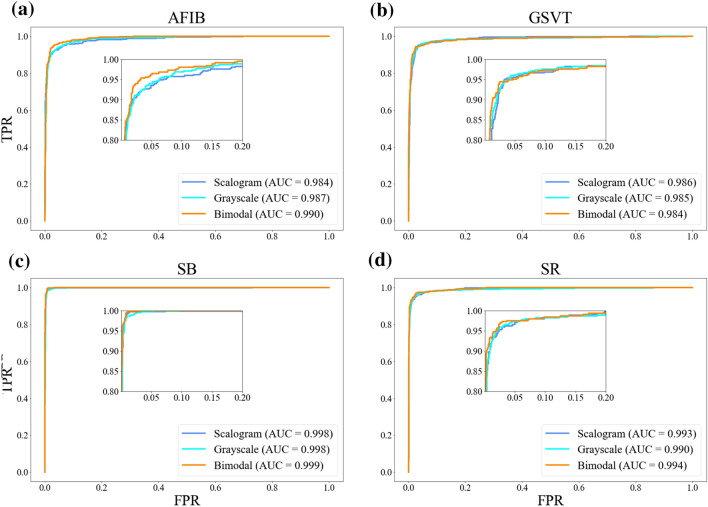
Comparison of the AUCs among grayscale image, scalogram, and bimodal models for lead II. TPR, true positive rate; FPR, false positive rate. (**a**) AFIB class (**b**) GSVT class (**c**) SB class (**d**) SR class.

In addition, we compared the diagnostic performance of the bimodal CNN model with single Inception-v3 model which was fine-tuned with grayscale images alone and with scalograms alone. As described in Table 3, a single Inception-v3 model which was fine-tuned with grayscale images obtained AUC (0.990), accuracy (93.85%), sensitivity (0.929), precision (0.934), and F1-score (0.931) in lead II. A single Inception-v3 model which was fine-tuned with scalograms achieved AUC (0.990), accuracy (94.09%), sensitivity (0.931), precision (0.935), and F1-score (0.932) in lead II. The AUC comparison among the grayscale image, scalogram, and bimodal models for four classes of ECG rhythms are depicted in Fig. 3. In order to compute the AUC and plot the ROC curve in Fig. 3, we took the One-vs-the-Rest (OvR) multi-class strategy. Only one class given at each stage was considered as a positive class and the remaining three classes were considered as a negative class. So, as shown in Fig. 3, we plotted the four ROC curves of AFIB, GSVT, SB, and SR and calculate AUC. On the other hand, the performance metrics in Table 3 represent the average values of the performance measures (AUC, SEN, PRE, F1-score) of each class calculated by the OvR multi-class strategy. To demonstrate the effectiveness of bimodal CNN, the model was compared with logistic regression, XGBoost and LSTM^23–25^. Table 4 summarizes the diagnostic performance and indicates that the bimodal CNN achieved the best results in AUC, accuracy, sensitivity, precision and F1-score.

**Table 3:** Diagnostic performance comparison among grayscale image, scalogram, and proposed CNN models in lead II (learning rate = 1e−4).

CNN models	AUC	ACC (%)	SEN	PRE	F1-score
Grayscale image CNN	0.990	93.85	0.929	0.934	0.931
Scalogram CNN	0.990	94.09	0.931	0.935	0.932
Bimodal CNN	0.992	95.08	0.942	0.946	0.944

**Table 4:** Diagnostic performance comparison among logistic regression, XGBoost, LSTM and the proposed bimodal CNN model.

	AUC	ACC (%)	SEN	PRE	F1-score
Logistic regression	0.975	88.00	0.865	0.873	0.868
XGBoost	0.981	90.26	0.888	0.898	0.892
LSTM	0.991	92.58	0.917	0.904	0.904
Bimodal CNN (Ensemble)	0.994	95.74	0.950	0.953	0.952

## Discussion

In this study, we developed a bimodal CNN model by co-training ECG grayscale images and scalograms for cardiovascular disease classification. The bimodal CNN method with two identical Inception-v3 backbones model showed AUC (0.992), accuracy (95.08%), sensitivity (0.942), precision (0.946), and F1-score (0.944). Ensemble model averaging the output probability information of all 12 leads achieved better AUC (0.994), accuracy (95.74%), sensitivity (0.950), precision (0.953), and F1-score (0.952).

As backbone models, we used Inception-v3 architecture which is a widely-used image recognition model. Inception-v3 adopts an inception module that allows multiple convolutional filters of different sizes to be concatenated into a new filter and cover receptive fields of different regions. The Inception module reduces computational complexity of the model by decreasing the number of trainable parameters^22^. In addition, this study showed that the proposed bimodal CNN architecture is applicable even when ResNet-50 and EfficientNet-B3 models are used as backbones. ResNet introduced skip connections to alleviate overfitting and performance degradation of deep neural networks. A skip connection is a feedforward network with a shortcut connection that adds new inputs to the network and yields new outputs^26^. EfficientNet scaled up CNNs in a more structured manner and used neural architecture search to design a new baseline network. The EfficientNet achieved better accuracy and efficiency than previous CNN models while being much smaller and faster on inference than existing CNN models^27^.

The two-dimensional ECG grayscale images are similar to those reviewed by cardiologists for CVDs classification. In other words, it can be said that the knowledge that cardiologists classify CVDs is learned by CNN model from ECG grayscale images. In this way, Jun et al. showed that 2D-CNN achieved good performance in classifying eight types of ECG signals from the MIT-BIH dataset^16^. Li et al. took ECG images as inputs, and performed arrhythmia classification using CNN and transfer learning to diagnose seven classes of arrhythmia^28^. In addition, Du et al. proposed a multi-label fine-grained network that consists of weakly supervised part discovery, spatial attention of discovered parts and recurrent label inference to detect abnormalities in CECG and DECG dataset^29^.

Although the method of converting one-dimensional ECG recordings to two-dimensional grayscale images showed reasonable performance, one-dimensional ECG recordings contain lots of noises such as baseline wandering effects, powerline interference, electromyographic noise and artifacts. Therefore, numerous preprocessing processes such as filtering and noise removal are required to ensure data integrity and improve model accuracy. To mitigate this problem, one-dimensional ECG recordings can be converted into scalograms or spectrograms which automate the noise filtering and feature extraction^30^. In this way, Madan et al. introduced a hybrid model called 2D-CNN-LSTM, which transformed the ECG signals into scalograms and then combined two learning models, CNN and LSTM. The results obtained were better than other conventional techniques^30^. In a similar way, Jeong & Lim converted 12-channel ECG recordings into time–frequency feature map through short-time Fourier transform (STFT)^17^. While we transformed one scalogram per one ECG channel, Jeong & Lim represented the information of 12 channels as one time–frequency feature map. They then applied a CNN model using time–frequency feature maps to classify eight types of arrhythmias and normal sinus rhythms.

As mentioned above, many studies have classified CVDs by converting ECG recordings to grayscale images or scalograms, but few studies have compared the two transformation methods. A comparison of the diagnostic performance of CVDs classification for each ECG lead based on the two transformation methods is described in the supplementary materials (Table S5). For most of the leads, the scalogram transformation method showed better accuracy in classifying CVDs than the grayscale image transformation. This is probably due to the fact that the scalogram shows noise robustness, as suggested by Madan et al.^30^. However, although scalograms performed better in this study, there is no guarantee that scalograms always outperform grayscale images.

Recently many studies have shown that the performance of multi-input deep learning approaches can be better than that of conventional single-input deep learning approaches. For example, Choi et al. developed a dual-input CNN that utilizes both anteriorposterior and lateral elbow radiographs for automatic detection of pediatric supracondylar fractures in conventional radiography^18^. The dual-input CNN provided an accurate diagnosis of pediatric supracondylar fracture comparable to radiologists. In addition, Rayan et al. used a series of three radiographs for the binomial classification of acute pediatric elbow radiograph anomalies^19^. They integrated a CNN and RNN to interpret an entire series of three radiographs together and obtained reasonable diagnostic performance.

As noted above, multi-input CNN models have been applied in various fields, but to the best of our knowledge, there are no studies dealing with ECG grayscale images and scalograms as bimodal inputs. Although there may be multiple input combinations of various ECG signals, we showed that the combination of ECG grayscale images and scalograms improves CVDs diagnostic performance.

For a comprehensive comparison, we investigated performance on two conventional machine learning algorithms (logistic regression and XGBoost) and LSTM. Statistical features were extracted from wavelet coefficients which are often used to classify cardiac arrhythmias to employ ECG signals to logistic regression and XGBoost. Wavelets are a popular tool for computational harmonic analysis with the advantage of providing localization in both the time (or space) domain and the frequency domain^10^. Comparing the logistic regression and XGBoost results with deep learning algorithms, in Table 4, LSTM and bimodal CNN showed better diagnostic performance than logistic regression and XGBoost. This is presumably because deep learning algorithms automatically extracted important feature vectors related to the target classes, whereas logistic regression and XGBoost used manually extracted features. As another comparison method, LSTM was applied to process one-dimensional ECG signals. LSTM is widely used for automatically interpreting ECG signals without the intervention of a cardiologist since they have the advantage of being able to learn well-distinguishable features inherent in raw ECG signals and to extract global time-dependent features related to time-varying dynamics through recurrent connection^11,31^. Despite the advantages mentioned above, bimodal CNN achieves better performance than LSTM as described in Table 4. We attribute these findings for two reasons. The first reason is the dual image input. The proposed model makes use of ECG grayscale images and scalograms. The second reason is the fine-tuning. In this research, we fine-tuned the proposed model using a pretrained model as opposed to LSTM trained from scratch. It is well known that fine-tuning a pretrained model on a target dataset can improve performance, speed up convergence, and is useful when limited amounts of labeled data are available^32^.

Although the proposed bimodal CNN model showed high potential for diagnosing CVDs, this study has several limitations. First, we used 12-lead ECG arrhythmia database collected by Chapman University and Shaoxing People’s Hospital to train, validate, and test the CNN model. Although that dataset contained more than 10,000 patients, still this study used only a single dataset. In machine learning and deep learning researches, the use of a single dataset could affect the generalization of training models. To mitigate this problem, we divided the dataset into training (64%), validation (16%), and testing (20%), but generalization issue may still be present. To address this issue, we should consider to use multiple large publicly available ECG datasets such as the recently published PTB-XL^33^. Second, in order to show the efficacy of the proposed bimodal model, we compared the bimodal model with logistic regression, XGBoost, LSTM, the grayscale image model and the scalogram model. However, it is necessary to show the comparison with the 1D-CNN model or transformers which are useful for time series data.

This study proposed a bimodal CNN algorithm that can classify cardiac arrhythmias using 12-lead ECG recordings. The bimodal CNN model, which converted the ECG recordings into grayscale images and scalograms, achieved reasonable results to classify AFIB, GSVT, SB and SR. Considering the ensemble model averaging the output probability information of all 12 leads, we expect that our bimodal CNN model will be of great help in diagnosing CVDs.

## Conclusion

In this study, we proposed a bimodal CNN model by co-training ECG grayscale images and scalograms for CVDs classification. Experiments were performed on a large public ECG database collected by Chapman University and Shaoxing People’s Hospital and the proposed model achieved better performance than logistic regression, XGBoost, LSTM and single CNN models for classifying CVDs. These results suggest that the bimodal CNN may be a promising method to improve the accuracy of CVDs diagnosis. Further experiments on other large ECG datasets are needed to demonstrate the generalization of the proposed bimodal CNN model.

## Materials and methods

### Study subjects and ECG recording preprocessing

This study employed a 12-lead ECG database collected by Chapman University and Shaoxing People’s Hospital (Shaoxing Hospital of the Zhejiang University School of Medicine) to validate the proposed bimodal CNN model^21^. This ECG database was recorded from 10,646 patients (5956 males) with 500 Hz sampling rates for 10 s. The ECG database contains 11 sets of heart rhythms that were labeled by professional physicians. A total of 10,588 ECG recordings were used in this study because some ECG recordings contained only zeros and some ECG lead values were missing. The following steps were implemented to preprocess the raw ECG signal as follows:

1. A Butterworth low-pass filter was used to reject frequencies above 50 Hz^34^,
2. The local polynomial regression smoother (LOESS) curve fitting was applied to remove the baseline wandering effect^35^,
3. The non-local means (NLM) were used to reduce residual noises^36^.

As shown in Table 1, some ECG rhythms such as AVRT and SAAWR rhythms contained a very small number of samples, which can lead to data imbalance problems. So, as suggested by Zheng^21^, the 11 rhythms were hierarchically merged into four ECG groups (AFIB, GSVT, SB, and SR). Details on numerical information and groups of the ECG recordings are described in Table 1.

### Machine learning and LSTM

To obtain the feature vectors of the ECG signal, we first calculated the wavelet coefficients of the ECG signal using a multi-level (5-level) 1-D discrete wavelet transform (Daubechies db6) for each lead. Various statistical feature vectors were then extracted from the wavelet coefficients, including the 5th, 25th, 75th and 95th percentiles, median, mean, standard deviation, variance, root mean square, zero and mean crossings as suggested in^37^. The extracted statistical feature vectors were fed as input to the machine learning algorithms.

Among machine learning algorithms, logistic regression and XGBoost were evaluated in this study. Logistic regression was implemented using Scikit-learn which is an open source machine learning library (https://scikit-learn.org/stable/index.html) and XGBoost was implemented by XGBoost Python Package (https://xgboost.ai/). Optimal hyperparameters of the logistic regression and XBGoost were obtained based on accuracy through exhaustive grid search. For logistic regression, the inverse of the regularization strength was explored in (1e-4, 1e-3, 1e-2, 1e-1, 1, 1e1, 1e2, 1e3, 1e4). For XGBoost, three hyperparameters were searched: n_estimators (number of trees), max_depth (maximum depth of a tree), eta (learning rate). The cross-product of n_estimators values in (100, 500), max_depth values ranging in (3, 5, 7, 9) and eta values in (0.01, 0.05) was thoroughly investigated.

There are many hyperparameters in LSTM, but in this study, the batch size, hidden size, dropout, and number of epochs were set to fixed values of 128, 128, 0.2, and 100, respectively. The training model was optimized with Adam optimizer ${\beta }_{1}=0.9$ and ${\beta }_{2}=0.999$. The learning rate and the number of layers, which are hyperparameters, were obtained by grid search. The search range for the learning rates was in (1e-3, 1e-4, 1e-5) and the number of layers was in (2, 3, 4). The hyperparameter with the highest accuracy in the validation data was selected as the optimal hyperparameter.

### One-dimensional ECG recordings to two-dimensional image transformation

In this study, we converted one-dimensional ECG recordings into two-dimensional grayscale images and scalograms since the proposed bimodal CNN model requires images as inputs. For grayscale images, one-dimensional ECG recordings were plotted as grayscale images with a black background and a white ECG signal. Then the grayscale images were saved as 300 × 300 pixels. In order to generate a scalogram, a continuous wavelet transform (CWT) is applied to the ECG recordings. CWT analyzes signals jointly in time and frequency and can provide more interpretable results than the STFT^38^. When converting the ECG recordings to scalograms, we used the ***cwt.m*** function of the Wavelet Toolbox in Matlab 2020a (https://www.mathworks.com). The converted scalogram was saved as a 300 × 300 pixel RGB image. Sample images of grayscale images and scalograms for the four groups of ECG recordings (AFIB, GSVT, SB, and SR) are shown in Fig. 1.

### Bimodal CNN model

The bimodal CNN model uses both grayscale images and scalograms as input images. Each different modality of input image was fed concurrently into two identical pretrained Inception-v3 backbone models. The input image was resized to 299 × 299 since the Inception-v3 has an image input size of 299 × 299. Instead of the Inception-v3 model, various CNN models can be used as the backbone model. The diagnostic performance for the ResNet-50 and EfficientNet-B3 as the backbone model was described in supplementary materials (Table S3, Table S4). CNN model can be divided into a feature extractor part and a classifier part. In the bimodal CNN model, features are extracted from the feature extractor part of the each pretrained Inception-v3 model. In Inception-v3, the feature map of the last inception module (Mixed_7c) of the feature extractor part has a size of 8 × 8 × 2048 dimensions. Average pooling of the feature map results in a 1 × 1 × 2048-dimensional vector, which is used as a feature vector in this study. As shown in Fig. 2, the feature vectors extracted from the ECG grayscale image and the feature vectors obtained from the scalogram are concatenated. The concatenated feature vectors result in 4096-dimensional feature vectors. The concatenated feature vectors are propagated to a classifier part consisting of fully connected layers and softmax activation function to classify the four ECG recording groups. The classifier part with fully connected layer and softmax activation function outputs prediction values in the range of 0–1 for the four labels. Since we replaced the classifier layers with a new classifier with four classification nodes (AFIB, GSVT, SB, and SR), the weights of the new classifier were initialized with random values. On the other hand, the weights of the convolution layers are initialized with the pretrained weights of the Inception-v3 model. Then, the weights of the feature extractor part of two identical Inception-v3 backbone models are individually fine-tuned and the weights of the classifier part are retrained from scratch using the training set of ECG grayscale images and scalograms by propagating errors backwards.

When fine-tuning the proposed model, the Adam optimizer (${\beta }_{1}=0.9$ and ${\beta }_{2}=0.999$) was used with initial learning rates (1e−4, 5e−5, 1e−5). Of the three learning rates, we adopted 1e-4 since the learning rate showed best accuracy on the validation set. The mini-batch size was set to 8 and the number of epoch was fixed to 30. The bimodal CNN model was developed with PyTorch framework^39^ on a computer with an Intel Xeon 2.2 GHz processor, a 26 GB RAM, and an NVIDIA Tesla P100 16 GB GPU.

### Performance measures

In order to evaluate the discriminative power of the bimodal CNN model, we calculated overall classification accuracy (ACC), sensitivity (SEN), precision (PRE), F1-score and the area under the receiver operating characteristic curve (AUC) for quantitative measurements. The AUC is the computed values of (1-specificity) and sensitivity^40^. The mathematical formulas for the above-mentioned measures are given as follows:

$$ACC = \frac{TP + TN}{{TP + FP + FN + TN}} \times 100\%$$

$$SEN = \frac{TP}{{TP + FN}}$$

$$PRE = \frac{TP}{{TP + FP}}$$

$$F1 - score = 2 \times \left( {\frac{SEN \times PRE}{{SEN + PRE}}} \right)$$

In the formulas, TP, FP, FN and TN are the abbreviations for the true positive, false positive, false negative and true negative, respectively.

## Supplementary Information

Supplementary Tables.

## Data Availability

The data used in this study are freely and publicly available. The Chapman University and Shaoxing People’s Hospital (Shaoxing Hospital of the Zhejiang University School of Medicine) dataset can be accessed at https://figshare.com/collections/ChapmanECG/4560497/2.
