# Supplementary material for: Bimodal CNN for cardiovascular disease classification by co-training ECG grayscale images and scalograms
Source: Sci Rep. 2023 Feb 20;13:2937. doi: 10.1038/s41598-023-30208-8 (PMC9941114; doi:10.1038/s41598-023-30208-8)
Supplement: Supplementary file 1 — Supplementary Tables. [file 41598_2023_30208_MOESM1_ESM.docx]

**Bimodal CNN for cardiovascular disease classification by co-training ECG grayscale images and scalograms**

Taeyoung Yoon, Daesung Kang*****

Department of Healthcare Information Technology, Inje University, Gimhae-si, Republic of Korea

Table S1. Diagnostic performance of bimodal CNN model for all leads (learning rate = 1e-5)

| Lead names | AUC | ACC(%) | SEN | PRE | F1-score |
| --- | --- | --- | --- | --- | --- |
| Lead I | 0.987 | 92.05 | 0.907 | 0.916 | 0.909 |
| Lead II | 0.99 | 93.8 | 0.928 | 0.931 | 0.929 |
| Lead III | 0.992 | 94.75 | 0.939 | 0.943 | 0.94 |
| aVR | 0.989 | 93.14 | 0.92 | 0.924 | 0.921 |
| aVL | 0.985 | 93.99 | 0.929 | 0.934 | 0.931 |
| aVF | 0.985 | 94.7 | 0.939 | 0.942 | 0.94 |
| V1 | 0.989 | 93.57 | 0.925 | 0.93 | 0.927 |
| V2 | 0.985 | 93.05 | 0.919 | 0.923 | 0.92 |
| V3 | 0.985 | 93.28 | 0.922 | 0.927 | 0.924 |
| V4 | 0.985 | 93.19 | 0.92 | 0.924 | 0.922 |
| V5 | 0.98 | 91.3 | 0.899 | 0.907 | 0.9 |
| V6 | 0.979 | 91.77 | 0.904 | 0.911 | 0.905 |
| Ensemble | 0.994 | 95.36 | 0.946 | 0.949 | 0.947 |

Table S2. Diagnostic performance of bimodal CNN model for all leads (learning rate = 5e-5)

| Lead names | AUC | ACC(%) | SEN | PRE | F1-score |
| --- | --- | --- | --- | --- | --- |
| Lead I | 0.989 | 93.19 | 0.921 | 0.927 | 0.923 |
| Lead II | 0.992 | 94.89 | 0.943 | 0.943 | 0.943 |
| Lead III | 0.991 | 94.37 | 0.935 | 0.940 | 0.937 |
| aVR | 0.990 | 94.70 | 0.938 | 0.943 | 0.94 |
| aVL | 0.985 | 93.95 | 0.929 | 0.933 | 0.931 |
| aVF | 0.987 | 94.65 | 0.938 | 0.943 | 0.940 |
| V1 | 0.990 | 94.23 | 0.934 | 0.937 | 0.935 |
| V2 | 0.991 | 94.18 | 0.933 | 0.935 | 0.934 |
| V3 | 0.984 | 91.63 | 0.902 | 0.911 | 0.904 |
| V4 | 0.987 | 92.19 | 0.909 | 0.913 | 0.910 |
| V5 | 0.982 | 91.91 | 0.905 | 0.913 | 0.908 |
| V6 | 0.982 | 91.82 | 0.906 | 0.912 | 0.907 |
| Ensemble | 0.994 | 95.70 | 0.950 | 0.952 | 0.951 |

Table S3. Diagnostic performance of bimodal CNN model when ResNet-50 was used as backbone model for all leads (learning rate = 1e-4)

| Lead names | AUC | ACC(%) | SEN | PRE | F1-score |
| --- | --- | --- | --- | --- | --- |
| Lead I | 0.990 | 93.00 | 0.919 | 0.926 | 0.921 |
| Lead II | 0.991 | 94.51 | 0.936 | 0.939 | 0.937 |
| Lead III | 0.987 | 93.24 | 0.928 | 0.924 | 0.925 |
| aVR | 0.991 | 93.85 | 0.932 | 0.931 | 0.931 |
| aVL | 0.986 | 93.09 | 0.920 | 0.924 | 0.921 |
| aVF | 0.990 | 94.32 | 0.936 | 0.937 | 0.936 |
| V1 | 0.990 | 93.57 | 0.926 | 0.930 | 0.927 |
| V2 | 0.983 | 93.42 | 0.923 | 0.928 | 0.925 |
| V3 | 0.987 | 94.23 | 0.932 | 0.935 | 0.934 |
| V4 | 0.985 | 93.52 | 0.924 | 0.930 | 0.926 |
| V5 | 0.983 | 92.76 | 0.916 | 0.922 | 0.918 |
| V6 | 0.987 | 92.67 | 0.915 | 0.920 | 0.917 |
| Ensemble | 0.992 | 95.36 | 0.946 | 0.949 | 0.947 |

Table S4. Diagnostic performance of bimodal CNN model when EfficientNet-B3 was used as backbone model for all leads (learning rate = 1e-5)

| Lead names | AUC | ACC(%) | SEN | PRE | F1-score |
| --- | --- | --- | --- | --- | --- |
| Lead I | 0.990 | 93.38 | 0.923 | 0.929 | 0.925 |
| Lead II | 0.993 | 95.41 | 0.946 | 0.951 | 0.948 |
| Lead III | 0.992 | 95.60 | 0.948 | 0.954 | 0.951 |
| aVR | 0.991 | 94.47 | 0.935 | 0.941 | 0.937 |
| aVL | 0.989 | 94.75 | 0.939 | 0.943 | 0.941 |
| aVF | 0.991 | 94.61 | 0.937 | 0.941 | 0.939 |
| V1 | 0.99 | 94.56 | 0.938 | 0.939 | 0.938 |
| V2 | 0.987 | 94.65 | 0.937 | 0.940 | 0.939 |
| V3 | 0.987 | 94.09 | 0.931 | 0.934 | 0.932 |
| V4 | 0.981 | 91.77 | 0.903 | 0.910 | 0.906 |
| V5 | 0.984 | 92.57 | 0.913 | 0.920 | 0.915 |
| V6 | 0.982 | 93.24 | 0.921 | 0.927 | 0.923 |
| Ensemble | 0.994 | 95.70 | 0.949 | 0.952 | 0.951 |

Table S5. Diagnostic performance comparison between ECG grayscale image based CNN model and scalogram based CNN (learning rate = 1e-4)

SCALO, scalogram; GRAY, grayscale image.

| Lead names | AUC | | ACC | | SEN | | PREC | | F1-score | |
| --- | --- | --- | --- | --- | --- | --- | --- | --- | --- | --- |
|  | SCALO | GRAY | SCALO | GRAY | SCALO | GRAY | SCALO | GRAY | SCALO | GRAY |
| Lead I | 0.984 | 0.985 | 92.53 | 92.15 | 0.912 | 0.907 | 0.920 | 0.920 | 0.915 | 0.912 |
| Lead II | 0.990 | 0.990 | 94.09 | 93.85 | 0.931 | 0.929 | 0.935 | 0.934 | 0.932 | 0.931 |
| Lead III | 0.989 | 0.991 | 94.51 | 93.99 | 0.935 | 0.929 | 0.940 | 0.934 | 0.937 | 0.931 |
| aVR | 0.991 | 0.987 | 94.18 | 93.24 | 0.932 | 0.924 | 0.937 | 0.924 | 0.933 | 0.924 |
| aVL | 0.985 | 0.985 | 93.19 | 93.19 | 0.920 | 0.922 | 0.925 | 0.925 | 0.922 | 0.923 |
| aVF | 0.991 | 0.909 | 94.13 | 93.28 | 0.932 | 0.923 | 0.938 | 0.925 | 0.934 | 0.924 |
| V1 | 0.991 | 0.990 | 94.70 | 94.04 | 0.938 | 0.930 | 0.943 | 0.935 | 0.940 | 0.932 |
| V2 | 0.988 | 0.981 | 94.32 | 92.43 | 0.934 | 0.913 | 0.938 | 0.918 | 0.936 | 0.914 |
| V3 | 0.986 | 0.984 | 93.80 | 92.81 | 0.928 | 0.917 | 0.932 | 0.920 | 0.930 | 0.918 |
| V4 | 0.983 | 0.985 | 91.44 | 92.86 | 0.906 | 0.917 | 0.910 | 0.923 | 0.905 | 0.918 |
| V5 | 0.984 | 0.983 | 93.38 | 92.34 | 0.923 | 0.912 | 0.927 | 0.914 | 0.925 | 0.913 |
| V6 | 0.986 | 0.987 | 92.90 | 92.62 | 0.917 | 0.914 | 0.924 | 0.919 | 0.920 | 0.916 |
| Ensemble | 0.992 | 0.992 | 95.13 | 95.13 | 0.943 | 0.943 | 0.947 | 0.947 | 0.945 | 0.945 |
